# ‘M-TRACK’ (mobile phone reminders and electronic tracking tool) cuts the risk of pre-treatment loss to follow-up by 80% among people living with HIV under programme settings: a mixed-methods study from Gujarat, India

**DOI:** 10.1080/16549716.2018.1438239

**Published:** 2018-02-27

**Authors:** Kedar Mehta, Ajay M. V. Kumar, Sudhir Chawla, Paragkumar Chavda, Kalaiselvi Selvaraj, Kalpita S. Shringarpure, Dipak M. Solanki, Pramod B. Verma, B. B. Rewari

**Affiliations:** ^a^ Department of Community Medicine, GMERS Medical College, Vadodara, India; ^b^ Centre for Operational Research, International Union Against Tuberculosis and Lung Disease (The Union), Paris, France; ^c^ Centre for Operational Research, International Union Against Tuberculosis and Lung Disease (The Union), New Delhi, India; ^d^ Care, Support and Treatment Department, Gujarat State AIDS Control Society, Ahmedabad, India; ^e^ Department of Community Medicine, Pondicherry Institute of Medical Sciences, Puducherry, India; ^f^ Department of Preventive and Social Medicine, Medical College, Baroda, India; ^g^ Research Department, World Health Organization, New Delhi, India

**Keywords:** pre-treatment attrition, initial default, SORT IT, operational research, mHealth

## Abstract

**Background**: In 2016, the National AIDS Control Programme (NACP) in Gujarat, India implemented an innovative intervention called ‘M-TRACK’ (mobile phone reminders once every week for four weeks after diagnosis and electronic patient tracking tool) to reduce pre-treatment loss to follow-up (LFU) among people living with HIV (PLHIV) in Vadodara district while other districts received standard of care.

**Objectives**: To assess the effectiveness of M-TRACK in reducing pre-treatment LFU (proportion of diagnosed PLHIV not registering for HIV care by four weeks after diagnosis) and to explore the implementation enablers and challenges from health care providers’ and PLHIV perspective.

**Methods**: An explanatory mixed-methods study design was used wherein the quantitative phase (cohort study with two groups: Vadodara district exposed to M-TRACK and Rajkot district as unexposed) was followed by a qualitative phase (descriptive study involving group interview with 16 health care providers, personal interviews with two programme managers and telephonic interviews with 16 PLHIV). Data were collected during October 2016 to February 2017.

**Results**: During the pre-M-TRACK period (July–September 2016), the LFU proportion was similar [13% (25/191) in Vadodara; 15% (21/141) in Rajkot (*p = *0.8)]. During the M-TRACK period (October–December 2016), LFU decreased to 4% (9/209) in Vadodara (exposed), whereas it remained similar at 16% (18/113) in Rajkot (unexposed) district (*p = *0.02). PLHIV exposed to M-TRACK had an 80% lower risk of LFU (aRR 0.2; 95% CI: 0.1–0.5) compared with standard care, after adjusting for socio-demographics, time and clustering at district level. During interviews, M-TRACK was welcomed by both PLHIV and the counsellors. The latter felt it saved time by obviating the need for home visits and helped in documentation. Inconvenience of using landline phone available at the health facility, lack of budgets for reimbursement of mobile call expenses and internet connectivity problems were the key implementation challenges.

**Conclusion**: M-TRACK was highly effective in reducing the gap between diagnosis and treatment. It may be considered for scale-up after addressing the challenges noted.

## Background

The Joint United Nations Programme on HIV/AIDS (UNAIDS) reported that 36.7 million people were living with HIV (PLHIV) globally in 2015 [1]. India ranks third globally, with about 2.1 million PLHIV in 2015 as estimated by National AIDS Control Organization (NACO) [2]. As per the Sustainable Development Goals, it is envisioned to end the global HIV/AIDS epidemic by 2030 [3]. To achieve this, UNAIDS and the World Health Organization (WHO) have set an ambitious 90–90–90 target for countries [diagnosing 90% of estimated PLHIV, treating 90% of diagnosed using antiretroviral therapy (ART) and achieving viral suppression in 90% of treated] [4]. In terms of absolute numbers, this target translates to 33 million to be diagnosed, 30 million to be treated and 27 million to be virally suppressed by 2020. However, at the end of 2015, only 18.2 million were receiving ART, thus leaving a gap of about 40% from the target [5].

This gap between diagnosis and treatment (pre-treatment loss to follow-up) exists in many countries, though varying in its magnitude and ranging from 20% in India to 28% in United States and 45% in South Africa [6–8]. Analysis of routinely reported aggregate programme data in Gujarat (a state in Western India) indicates a gap of about 16% between the number of PLHIV diagnosed and the number registered for care at ART centres. Some of the common reasons for pre-treatment loss to follow-up (LFU) include lack of risk perception and feeling of wellness among PLHIV, difficulties in transportation, nondisclosure, stigma, fear of discrimination, poor geographical reach and financial hurdles [8–11].

Several interventions have been undertaken by the National AIDS Control Programme in Gujarat to address the economic constraints faced by the PLHIV, which include reimbursing the transport costs for visits to the ART centre and linking PLHIV to other social welfare benefits. Despite these measures, the gap between diagnosis and treatment still persists.

Thanks to the telecommunication revolution in India, the total number of mobile phone subscribers stood at 1.08 billion by the end of 2016 [12], and most patients now have access to mobile phones. In general, mHealth interventions using short text messages and/or phone call reminders have been effective in improving health-related outcomes, despite some challenges [13,14].

Previous studies in the HIV field have focussed on the effect on mHealth in improving treatment adherence, patient compliance to clinic appointments and follow-up care, prevention of mother to child transmission and early infant diagnosis, education and motivational behaviour change [11,14]. However, there have been no studies examining the effect of mobile reminders on reducing pre-treatment LFU among PLHIV. Further, there is limited information about the effectiveness and challenges of implementing mobile phone reminders in routine programme settings.

To strengthen the linkage of diagnosed PLHIV to treatment, the Gujarat State AIDS Control Society (GSACS) implemented an electronic tracking system coupled with mobile phone call reminders (M-TRACK) in Vadodara district of Gujarat from October 2016 on a pilot basis. Since M-TRACK was implemented only in one district and not in others, it provided us with an opportunity to assess the effectiveness of intervention at programmatic level (which required quantitative research methods) and to understand the implementation challenges (which required qualitative research methods).

Hence we planned to undertake a mixed-methods study with the following objectives: (1) to assess whether M-TRACK was effective in reducing pre-treatment LFU among PLHIV diagnosed in two selected districts of Gujarat [Vadodara (district exposed to M-TRACK) and Rajkot (district unexposed to M-TRACK)] during July–December 2016; and (2) to understand the enablers and challenges in implementation of M-TRACK from the health care providers’ and PLHIV perspective.

## Methods

### Study design

This was an explanatory mixed-methods study design wherein the quantitative phase (cohort study) was followed by a qualitative phase (descriptive study) [15].

### Study setting

The study was conducted in two selected districts out of 33 districts in the Gujarat State in Western India. M-TRACK was implemented by the National AIDS Control Programme in Vadodara district. We chose Rajkot district for comparison, as it had similar rates of pre-treatment LFU at baseline compared with Vadodara.

There are 19 HIV testing centres and two ART centres in Vadodara district, and 14 HIV testing centres and two ART centres in Rajkot district. Every client attending the HIV testing centre is counselled about the need for, and offered, HIV testing. If a client is found to be HIV-positive, post-test counselling is carried out, and the client is referred to the nearest ART centre for clinical and immunological assessment and treatment. All services including diagnosis and treatment are given free of charge for the PLHIV. While there is information about aggregate numbers of PLHIV diagnosed and registered at ART centres, there is no individual patient-wise tracking and cohort-wise assessment and reporting.

### M-TRACK intervention

M-TRACK consisted of two components: (1) electronic patient-wise tracking and (2) weekly mobile phone reminders.

First, all the staff at HIV testing centres and ART centres were trained in implementing M-TRACK, the structured content of the telephonic call and the data-collection procedure before the start of the intervention.

At the HIV testing centre, every HIV-infected client was asked if it was acceptable to him/her to be contacted over phone. If agreed, the client was requested to provide his/her mobile phone number (or phone number of a contact), and its functionality was tested on site by the counsellor. The counsellor also shared his/her mobile number and the official phone number of the hospital with the PLHIV to contact the former in case of any difficulties to access ART centre. This provided an opportunity for free exchange of information, for PLHIV to seek clarifications about HIV, treatment and service delivery, and for counsellors to share information on social welfare benefits.

#### Electronic patient-wise tracking

This involved entering the details of PLHIV into a specially designed Google spreadsheet or MS Excel spreadsheet, which was shared virtually between staff of the HIV testing centre and ART centre only. This spreadsheet consisted of two parts. The first part included demographic and clinical details, which were filled by staff of HIV testing centre soon after diagnosis. The second part included details about registration at ART centre, which was to be checked periodically (once a week) and filled by the ART centre staff.

#### Mobile phone reminders

**Figure 1.**
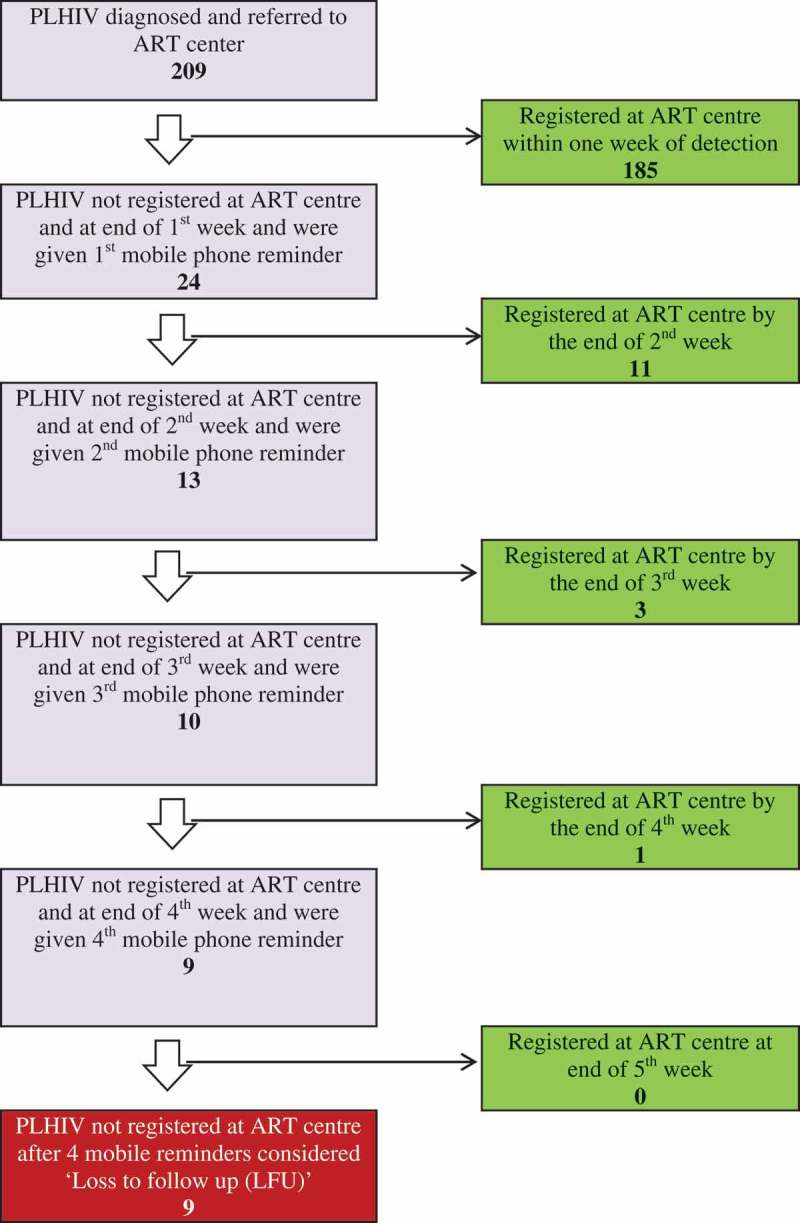
Effect of *M-TRACK* implementation on ART registration in Vadodara district among PLHIV diagnosed during October 2016 to December 2016 (*N* = 209). M-TRACK = Mobile phone reminders and electronic tracking tool; PLHIV = people living with the human immunodeficiency virus; ART = antiretroviral therapy.

The counsellor at HIV testing centre made phone calls to the PLHIV (using the official landline telephone available at the health facility or their personal mobile phones) to determine whether they have reached the ART centre and registered for care. The telephone call was repeated once every week until ART registration or 4 weeks of diagnosis, whichever was earlier. If the PLHIV replied that s/he had indeed reached the ART centre, this was validated with the records at the ART centre. If the PLHIV was not registered at the ART centre after the fourth reminder, s/he was considered ‘lost-to-follow-up’ (LFU) (Figure 1).

In Rajkot (unexposed district), the current standard of care was given, and the diagnosed PLHIV was contacted by telephone only after four weeks of diagnosis to ascertain their registration at ART centre, and if not registered then that PLHIV was considered ‘lost to follow-up’.

### Study population and study period

#### Quantitative

All PLHIV diagnosed in HIV testing centres of Vadodara (exposed) and Rajkot (unexposed) districts between July and December 2016 were included in the study. The period from July to September 2016 was considered a ‘pre M-TRACK period’ and October–December 2016 was considered an ‘M-TRACK period’.

#### Qualitative

Health care providers (counsellors and programme managers) involved in implementation of M-TRACK and PLHIV who received mobile phone reminders constituted the study population.

The data collection was carried out from October 2016 to February 2017.

### Data variables, sources of data and data collection

#### Quantitative

Data were collected using a structured data collection proforma in both districts and for both pre-M-TRACK and M-TRACK periods (Supplement 1). Data on demographic and clinical characteristics (age, gender, education, marital status, occupation, risk behaviour type, baseline CD4 count, WHO Clinical staging and availability of phone) were captured. In addition, for the Vadodara district (exposed to M-TRACK), additional details about the outcomes of the telephonic call were captured. Data entered by staff of HIV testing centres and ART centres were randomly checked for at least four HIV testing centres in a month by the investigators for its completeness and accuracy.

#### Qualitative

We conducted a group interview (GI) with the counsellors at a meeting room in the district health office. GI was conducted by two independent researchers, PC (moderator) and KM (record keeper) (male medical doctors, trained and experienced in qualitative research), who were not part of the team implementing M-TRACK. All 16 counsellors working in the M-TRACK exposed district participated. Only the counsellors and researchers were present in the room to ensure that participants shared their views freely. GI was conducted in local vernacular language and audio-recorded after taking informed consent. A discussion guide was used to explore the enabling factors, challenges and suggestions for improvement in M-TRACK implementation (Supplement 2). Field notes were prepared by the record keeper during the discussion. In addition, two key informants (state programme managers) were interviewed face to face at their respective office spaces in English language using an interview guide by two researchers, PC (interviewer) and KM (record keeper) (Supplement 3). Verbatim notes were taken during the interview. After the interview was over, the summary of the interviews was read back to ensure participant validation.

Efforts were made to contact and interview all the PLHIV who received mobile phone reminders. PLHIV who consented were interviewed by KM (trained in qualitative research) using an interview guide over telephone, in Hindi, Gujarati or English as preferred (Supplement 4). Among PLHIV who had not returned to care despite mobile phone reminders, reasons for the same were explored.

### Data entry and analysis

#### Quantitative

Data were analysed using EpiData (version 2.2.2.183, EpiData Association, Odense, Denmark) and Stata (version 11.0, College Station, TX: StataCorp LP). Our key outcome was pre-treatment LFU. The difference in the LFU before and after M-TRACK was assessed using a chi-square test. To assess if M-TRACK was independently associated with reduction on LFU, we adjusted for socio-demographic characteristics of PLHIV (with a *p* value of <0.1 in unadjusted analysis), time period and district-level clustering using a generalized estimating equation model (Poisson regression). We were not able to adjust for CD4 count and WHO clinical staging, as the information with respect to these variables was available only for PLHIV who were registered for HIV care at ART centres. We calculated the adjusted relative risk with 95% CI to assess the magnitude of association. A *p* value of <0.05 was considered statistically significant.

#### Qualitative

The GI and interviews were transcribed on the same day by KM based on field notes and audio recordings. A descriptive content analysis by manual coding was carried out by three independent trained researchers (KM, PC and KS) to generate various categories or themes under the broad topics: enablers, challenges and suggested solutions [15,16]. It was reviewed by other investigators (AMK and KSS) to reduce subjectivity in analysis. Any differences between the researchers were resolved by discussion [15]. A framework analysis was carried out to explore the enablers to M-TRACK implementation from health care providers and PLHIV perspective [17]. Enablers commonly listed by both PLHIV and providers were represented in a table. The findings have been reported using ‘Consolidated Criteria for Reporting Qualitative Research’ (COREQ) guidelines [18].

## Results

### Quantitative

#### Socio-demographic and clinical characteristics

There were 400 and 254 PLHIV diagnosed in Vadodara and Rajkot districts respectively during July–December 2016. PLHIV in the two districts were similar to each other with respect to most socio-demographic and clinical factors (such as sex, marital status, occupation, risk behaviour, availability of mobile phone and CD4 counts), except age, educational status and WHO clinical staging (Table 1). The mean age of PLHIV in Vadodara was 36 years, marginally lower than the mean age in Rajkot (38 years). About 33% of PLHIV had studied up to secondary school or higher in Vadodara compared with 12% in Rajkot. The proportion of PLHIV in stage 3 and 4 was higher in Vadodara (39%) compared to Rajkot (27%). About two-thirds of PLHIV were males, and about 80% were employed in both districts. More than 90% of PLHIV had mobile phones in both the districts.

**Table 1.** Clinical and socio-demographic profile of all PLHIV diagnosed at HIV testing centres from two selected districts (Vadodara and Rajkot), Gujarat, India, from July 2016 to December 2016.

	Vadodara (exposed)		Rajkot (unexposed)		
Variable	Number	Percentage	Number	Percentage	*p*
Total	400	(100)	254	(100)	
Age group (in years)					0.02
0–25	80	(20)	41	(16)	
26–35	138	(35)	67	(26)	
36–45	108	(27)	91	(36)	
≥46	74	(18)	55	(22)	
Gender					0.5
Male	263	(65)	177	(70)	
Female	135	(35)	76	(30)	
Transgender/Transsexual	2	(<1)	1	(<1)	
Education^a^					<0.01
Illiterate	90	(23)	60	(24)	
Primary	178	(45)	162	(64)	
Secondary	111	(28)	26	(10)	
Graduate and above	21	(5)	6	(2)	
Marital status					0.2
Married	255	(64)	147	(58)	
Single	70	(18)	46	(18)	
Divorced/Separated	32	(8)	32	(13)	
Widowed	43	(11)	29	(11)	
Occupation					0.06
Employed	306	(77)	211	(83)	
Unemployed	94	(23)	43	(17)	
Type of risk behaviour					0.1
Heterosexual	372	(93)	227	(89)	
Homosexual	6	(2)	10	(4)	
Others^b^	22	(5)	17	(7)	
Availability of phone (mobile)	371	(93)	241	(95)	0.4
CD4 count^c^/mm^3^ Median (IQR)	264	(120–439)	224	(101–356)	0.1
WHO clinical stage^c^					<0.01
Stage 1	167	(46)	92	(43)	
Stage 2	57	(16)	64	(30)	
Stage 3	75	(21)	35	(16)	
Stage 4	64	(18)	24	(11)	
Not recorded	3	(1)	0	(0)	

% column percent. ^a^Education status of PLHIV has been classified as: illiterate – not able to read and write; primary – studied until the 1st to 7th standard of schooling; secondary – 8th to 12th standard of schooling; graduate and above – attended college for higher studies. ^b^Others include history of blood transfusion, history of infected syringe use, parent to child. ^c^ *N* = 366 for Vadodara, *N* = 215 for Rajkot (Information on CD4 count, WHO staging and time to registration available only for those who registered at ART centre). HIV = human immunodeficiency virus; PLHIV = people living with the human immunodeficiency virus; ART = antiretroviral therapy; WHO = World Health Organization; IQR = interquartile range; M-TRACK = mobile phone reminders and electronic tracking tool.

#### Phone-call reminders

The number of PLHIV who had to be given phone call reminders and the number who got registered as a result after first, second, third and fourth reminders are shown in Figure 1. Of 209 PLHIV diagnosed in Vadodara and exposed to M-TRACK, 185 (88%) got registered within a week of diagnosis without the need for a reminder. Of the remaining 24, nearly half got registered after the first reminder itself. Only nine PLHIV did not register for care even after four reminders and were considered LFU.

#### Effectiveness of M-TRACK intervention

**Figure 2.**
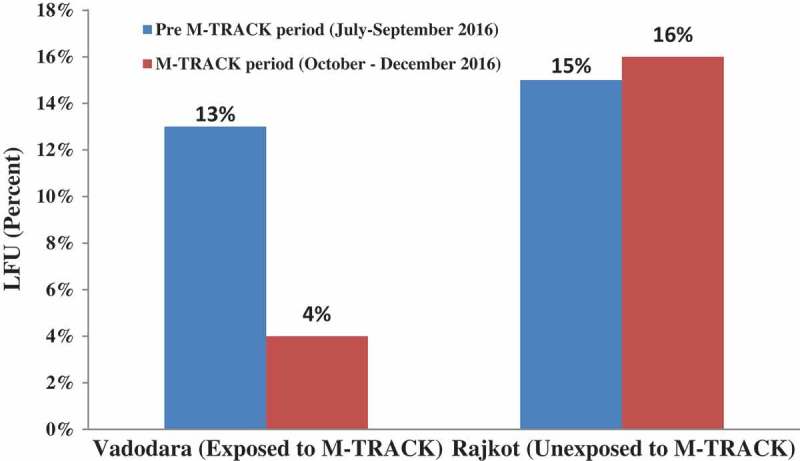
Effect of *M-TRACK* implementation on pre-treatment loss to follow-up among PLHIV diagnosed in Vadodara (exposed) and Rajkot (unexposed) districts of Gujarat, India, during July–December 2016. Pre-M-TRACK χ^2^ = 0.09 (*p* = 0.75) M-TRACK χ^2^ = 4.75 (*p* value = 0.02). PLHIV = people living with the human immunodeficiency virus; M-TRACK = mobile phone reminders and electronic tracking tool; LFU = loss to follow-up.

During the pre M-TRACK period, the pre-treatment LFU was similar in both districts – 13% (25/191) in Vadodara district and 15% (21/141) in Rajkot district (*p* value: 0.8). During the M-TRACK period, pre-treatment LFU decreased to 4% (9/209) in Vadodara (exposed) whereas it remained similar at 16% (18/113) in Rajkot (unexposed) district (*p* value: 0.02) (Figure 2).

PLHIV exposed to M-TRACK had an 80% lesser risk of pre-treatment LFU (aRR = 0.2 with 95% CI: 0.1–0.5, *p* value: 0.001) compared with standard care, after adjusting for socio-demographic factors, time period and clustering at district level in multivariable analysis (Table 2). The other factor associated with LFU was education level of PLHIV, and those who completed secondary education had a 60% lower risk of pre-treatment LFU (aRR = 0.4) relative to PLHIV who were illiterate.

**Table 2.** Multivariable analysis showing the effect of M-TRACK implementation on pre-treatment loss to follow-up (after adjusting for socio-demographic factors, time and clustering at district level) among PLHIV diagnosed in two selected districts of Gujarat, India during July–December 2016.

Variable	Total *N* = 654	LFU *N* = 73	(%)	RR (95% CI)	aRR (95% CI)
M-TRACK					
Yes	209	9	(4)	0.3 (0.1–0.6)	0.2 (0.1–0.5)^b^
No^a^	445	64	(14)	Ref	Ref
Age group (in years)					
0–25^a^	121	15	(12)	Ref	Ref
26–35	205	25	(12)	0.9 (0.5–1.8)	0.9 (0.5–1.9)
36–45	199	21	(11)	0.8 (0.5–1.6)	0.7 (0.4–1.5)
46–90	129	12	(9)	0.7 (0.3–1.5)	0.6 (0.3–1.4)
Gender					
Male	440	52	(12)	0.8 (0.5–1.3)	0.9 (0.5–1.6)
Female^a^	211	21	(10)	Ref	Ref
Education					
Illiterate^a^	150	24	(16)	Ref	Ref
Primary	340	37	(11)	0.7 (0.4–1.1)	0.6 (0.3–1.0)
Secondary	137	9	(6)	0.4 (0.2–0.8)	0.4 (0.2–0.9)^b^
Graduate and above	27	3	(11)	0.7 (0.2–2.1)	0.8 (0.2–2.7)
Marital status					
Married^a^	402	47	(12)	Ref	
Single	116	14	(12)	1.0 (0.6–1.8)	
Divorced/separated	64	6	(9)	0.8 (0.4–1.8)	
Widowed	72	6	(8)	0.7 (0.3–1.6)	
Occupation					
Employed	517	61	(12)	1.3 (0.7–2.4)	1.2 (0.5–2.5)
Unemployed^a^	137	12	(8)	Ref	Ref
Type of risk behaviour					
Heterosexual^a^	599	65	(11)	Ref	Ref
Homosexual	16	3	(19)	1.7 (0.6–4.9)	1.7 (0.5–5.7)
Others	39	5	(13)	1.2 (0.5–2.7)	0.9 (0.3–2.6)
Period^c^					
Pre M-TRACK period	332	46	(14)	0.6 (0.4–0.9)	1.2 (0.7–2.0)
M-TRACK period^a^	322	27	(8)	Ref	Ref

% row percent. ^a^Ref = Reference. ^b^Statistically significant. ^c^Period – Pre M-TRACK (July–September 2016) and M-TRACK period (October–December 2016). LFU = loss to follow-up (PLHIV not registered within 5 weeks of diagnosis); PLHIV = people living with the human immunodeficiency virus; RR = relative risk; aRR = adjusted relative risk; CI = confidence interval; M-TRACK = mobile phone reminders and electronic tracking tool.

### Qualitative

A total of 18 health care providers were interviewed. This includes all the 16 counsellors working in the HIV-testing centres (100% response rate) and two programme managers working at state level. We tried to contact all the 24 PLHIV who received mobile phone reminders, but were successful in reaching out to 16 PLHIV only (the remainder either were not reachable or declined to participate). Of 15 PLHIV registered for care, 12 PLHIV (seven males; five females; mean age 32 years) were interviewed. Among nine PLHIV who did not register, only four were interviewed (two males; two females; mean age 38 years).

#### Enablers to M-TRACK implementation

The enablers to M-TRACK from the perspective of PLHIV and health care providers are summarized in Table 3. Most enablers were commonly identified by both PLHIV and providers and included (1) helped in rapport building, (2) gave an opportunity to exchange information on not only treatment services but also on other government welfare schemes, (3) helped in maintaining patient confidentiality and (4) increased the client’s confidence in the public health care system. In addition, providers mentioned that M-TRACK made it easier to follow-up and track PLHIV, helped in documentation, saved time by reducing the need for home visits and helped in better planning and scheduling of home visits, when required. Counsellors mentioned that they felt motivated on hearing testimonies of satisfied clients.

**Table 3.** Enabling factors for M-TRACK implementation as perceived by PLHIV and health care providers in Vadodara district, Gujarat, India during October–December 2016.

Themes	Categories	PLHIV	Health care providers
Clients-related	Acts as two-way channel of communication		√
	Helps in rapport building	√	√
	Opportunity to seek/provide timely information on treatment services, HIV testing of family members and other issues such as travel allowance details and benefits offered by other government welfare schemes	√	√
	Client feels cared for by the provider	√	√
	Increases the client’s confidence in the public health system	√	√
	Helps maintain patient confidentiality and perceived by counsellors to be better than home visits	√	√
Counsellors-related	Helps in record keeping and documentation		√
	Points missed during counselling at health facility can be covered during the phone call	√	√
	Minimizes the need for home visits by counsellors	√	√
	Helps in planning the time of home visits and saves time owing to patient unavailability	√	√
	Easy to follow up clients and obtain information in an organized manner and in a short time		√
	Hearing from satisfied clients motivates individual to work better		√

HIV = human immunodeficiency virus; PLHIV = people living with the human immunodeficiency virus; M-TRACK = mobile phone reminders and electronic tracking tool.

#### Challenges to M-TRACK implementation from health care providers’ perspective

Challenges were grouped into five broad themes and 16 codes. Challenges (along with verbatim quotes of health care providers) from the health care providers’ perspective are described below (see Supplements 5 and 6 for additional verbatim quotes).

#### Theme I: client (PLHIV)-related challenges

One challenge was related to the timing of the phone call. During GI, health care providers mentioned that a small proportion of PLHIV (estimated to range from 25 to 30%) preferred to receive calls after office hours or on holidays.

Many times patient may not be able to talk during office hours and they suggest us to call in the evening. Now, we cannot come to the office to call our clients in the evening. (male counsellor, 28 years)

A few counsellors reported having experienced verbal abuse by PLHIV for calling them and threats to lodge police complaints against them. Some also mentioned language barrier as a hindrance in communicating with PLHIV from other states.

In instances where PLHIV did not own a mobile phone (especially those from tribal areas), family members had to be contacted, which had a risk of breach in the patient confidentiality.

When the patient has to be contacted through someone else in the family (when patient does not own the phone), we have to first explain in detail to reach the client. In such cases, we may have to break the confidentiality. (female counsellor, 32 years)

#### Theme II: staff-related challenges

Some counsellors reported disturbances in their personal life because they had to receive calls by PLHIV during night time after office hours and on holidays.

When patient gets our mobile number, they call us any time, even at night, which creates problems in our family. (male counsellor, 27 years)

Many counsellors at HIV counselling and testing centres reported that they did not receive timely and regular updates from the staff of the ART centres regarding the status of registration of PLHIV at ART centre.

#### Theme III: phone-related challenges

The majority (13/16) of counsellors who participated in GI mentioned that they were not able to use a landline phone available at the health facility to call PLHIV and had to resort to using their personal mobile phones. The reasons mentioned by them for not using landline phone were: non-functional landline phone, discomfort in counselling PLHIV regarding sensitive issues in the presence of other office staff, risk of a breach in PLHIV confidentiality (when they address PLHIV by their names), disturbance due to ambient noise (in office) and unfeasibility to use after office hours.

There is always ambient noise. The office staff might be talking aloud or even laughing over their internal discussion. Because of this, patient might feel stigmatized. Overall this makes the counselling difficult. (female counsellor, 35 years)

#### Theme IV: M-TRACK tool-related challenges

All counsellors reported that they were not able to use the Google spreadsheet owing to issues related to internet connectivity and lack of a dedicated computer. Thus, real-time data entry was not possible, and so they used MS Excel format, filled it offline and shared it with the ART centre via email as and when internet connectivity was available.

Not all centres have internet connection. At places where the connection is available, usually there are problems of connectivity. So we are not able to enter data in real time in the Google spreadsheet. So, we complete the Excel sheet [offline] and send it to ART centre [by email]. (male counsellor, 29 years)

#### Theme V: programme-related challenges

The key programmatic challenge was a lack of dedicated budgets within the NACP for reimbursing call charges and internet charges. The counsellors had to bear these costs themselves.

We should get the reimbursement of the call charges. Otherwise the motivation to work will be affected. (male counsellor, 32 years)

The other challenge perceived by the state programme manager was that it was difficult to sustain M-TRACK without regular monitoring and supervision from the district and state level.

Mobile phone reminders is a good approach for reducing LFU from ICTC [HIV testing centre] to ART center. But, at programmatic level, I feel it is difficult to sustain without regular follow-up mechanism from state level (GSACS). (Additional Project Director, Gujarat AIDS Control Society, 60 years)

#### Challenges from the perspective of PLHIV

All the 12 PLHIV (who registered for care after receiving phone-call reminders) interviewed were happy to receive phone-call reminders.

When I received call, the way they talked to me, I liked a lot. They explained me very well in the language and words which I can understand. He helped me for getting registered at ART centre. He also informed me about the to and fro travel money will be paid to me from centre. (PLHIV, female, 35 years)

They mentioned that they did not face any major challenge, barring a couple of them who had difficulties in receiving calls during office hours, in front of other colleagues. Three PLHIV preferred to receive short text messages or Whatsapp messages either alone or in addition to phone call reminders.

I think apart from calling a patient for registration and for medicines, they should also send messages daily for medicines so that patient doesn’t forget. Also some messages on what food to eat, what exercise to do and some basic health-related information should be given by them. (PLHIV, male, 28 years)

Solutions suggested by health care providers to address these challenges are summarized in Table 4. The key solutions included (1) provision of a dedicated mobile phone and reimbursing the call and internet charges; (2) obtaining prior consent specifically for the phone calls to avoid verbal abuse or threats by PLHIV; (3) requesting the PLHIV to avoid calling at night and holidays, unless there is an emergency; and (4) regular monitoring and supervision of the process by the district- and state-level supervisory staff.

**Table 4.** Solutions suggested by health care providers for M-TRACK implementation in Vadodara, Gujarat during October–December 2016.

Mobile phone and internet costs	● Provision of dedicated mobile phones to counsellors ● Reimbursing the costs of telephone call and internet use to counsellors
Consent and legal issues	● Consent for phone calls should be taken from the client during pre-test counselling ● Consent to contact the clients through the identified contacts (family members, caretakers or neighbours) should also be taken and documented ● There should be a mention in the job contracts of counsellors that they are authorized to call PLHIV over the phone ● Provision of protection to counsellor in case of legal action by PLHIV
Training for counsellor	● Counsellors should be trained for counselling in Hindi language (most widely spoken language in India) ● Counsellors should be trained to enter data on the Google spreadsheet using the mobile phone
Counselling the client	● Sharing the contact details of counsellor with the clients and asking them to contact, if required ● PLHIV need to be advised at the time of diagnosis that they should avoid calling outside office hours and during holidays, except in emergencies
Monitoring and supervision	● Development of a comprehensive software for ‘PLHIV – continuum of care’ from HIV testing centre to ART ● Regular monitoring on a daily basis by the district supervisor and on a weekly basis by the state officer

HIV = human immunodeficiency virus; PLHIV = people living with the human immunodeficiency virus; ICTC = Integrated Counselling and Testing Centre ART = anti-retroviral therapy; M-TRACK = mobile phone reminders and electronic tracking tool.

## Discussion

This is the first study assessing the feasibility and effectiveness of mobile phone reminders in reducing the pre-treatment LFU among PLHIV diagnosed in routine programme settings. While there are many studies including systematic reviews assessing the use of mobile health technology (mHealth) to address gaps in the cascade of HIV/AIDS care, most of them are focussed on improving health outcomes once the ART has been started but not addressing the gaps between diagnosis and treatment [14,19–23]. The interventions that have been tested to improve linkage to or retention in pre-ART care include point-of-care CD4 testing, providing food incentives, home-based ART initiation and health system interventions such as integration of antenatal services with ART care, adoption of WHO guidelines and introduction of a structured pre-ART care [20].

We found that M-TRACK was highly effective in reducing risk of pre-treatment LFU by 80%. This is excellent, considering it addresses the neglected area of pre-ART care. This success might be due to the combined effect of targeted messaging to PLHIV at the time of diagnosis (that they will be contacted by phone if not registered), rapport building between PLHIV and providers, enhanced patient tracking and weekly phone reminders. It will be difficult to tease out the impact of each of these components.

We consider that M-TRACK is feasible for the following reasons. First, nearly 95% of PLHIV have access to mobile phones in this setting. Second, the proportion of PLHIV requiring mobile phone reminders was only 12%. In absolute terms, only 24 PLHIV (56 phone calls) required to be reminded in a quarter in a district with 16 counsellors. This translates to about four phone calls per counsellor per quarter on average. Thus, M-TRACK does not pose any extra burden or workload for the counsellors. On the contrary, M-TRACK was welcomed by the counsellors, as they felt it saved time by obviating the need for home visits and helped in documentation. Some counsellors felt motivated hearing to the testimonies of satisfied clients over phone. Third, minimal training (three hours) was required for counsellors to understand M-TRACK and was integrated within the scheduled monthly meeting.

There were other advantages to M-TRACK. This included exchange of information on issues beyond HIV such as linkage to government welfare schemes. This was perceived by both clients and counsellors to increase the confidence of the client in the public health services. Additionally, M-TRACK enables cohort analysis, and thus it is easy to introduce an indicator on pre-treatment LFU in the quarterly report for monitoring and review.

However, there were some key challenges. These included inconvenience of using a landline phone available at the health facility, lack of provision for reimbursement of mobile call expenses and internet-connectivity problems. As suggested by the providers, programme budgets need to be reallocated to provide mobile phone and internet allowance to the counsellors. Another challenge was related to a lack of dedicated computer to fill up the Google spreadsheet. A possible solution for this could be to train the counsellors to use the mobile phone to enter the data online. These issues need to be addressed. Another challenge relates to possible breach in patient confidentiality during the process of telephonic contact, especially among PLHIV who do not have personal mobile phones and rely on the home telephone used by all family members. This was carefully considered, and all the counsellors were trained in using a structured content for the telephone conversation.

A systematic review has reported challenges related to funding, infrastructure and lack of clarity on the role of health systems in mHealth implementation [13]. Some reviews have noted potential challenges for scale-up and detailed the elements necessary for successful scale-up in developing countries [24,25]. These have to be factored in before planning a scale-up of M-TRACK across the state.

There are several strengths to this study. First, the setting created a ‘natural experiment’-like situation, providing us an opportunity to assess the effectiveness of intervention in the routine programme settings. Second, we conducted a robust analysis to assess the effectiveness of M-TRACK by adjusting all the possible confounders including PLHIV characteristics, time and district-level clustering. Third, since it was a mixed-methods study, we were able also to assess the enablers, challenges and solutions to address those challenges. These provided useful insights to assess feasibility, acceptability and scalability of M-TRACK. Finally, we adhered to STROBE guidelines for quantitative results reporting and COREQ guidelines for reporting the findings of the qualitative results [18,26].

There were several limitations to the study. First, we could not adjust for the effects of clinical parameters such as CD4 count and WHO clinical stage, as they correlated perfectly with pre-treatment LFU (among PLHIV with information on CD4 count/WHO stage, no one was LFU, and among PLHIV without the information, everyone was LFU). Second, these are early results, and we are not sure if the effects of this intervention will be sustained in the long term. Every novel intervention is accompanied by initial enthusiasm among all stakeholders, which may account for the early effectiveness. To determine whether the effect can be sustained, follow-up assessments for a longer period and sustained supervision and monitoring will be required.

In general, there is limited evidence on the cost-effectiveness of mHealth interventions [24], and we did not undertake a formal analysis of costs (of call charges, internet charges and other indirect costs). This could be a topic for future research.

## Conclusion

In conclusion, M-TRACK was feasible and effective in reducing the risk of pre-treatment LFU by 80% among PLHIV. If the identified challenges in the implementation of M-TRACK can be addressed, this intervention would have the potential to reduce the gap between diagnosis and treatment and achieve the second ‘90’ of the ambitious 90–90–90 target, and may be considered for scale-up by the national programme.

## Supplementary Material

Supplementary material

## Paper context

Previous research assessing the use of mobile phones in HIV/AIDS care has focussed on improving treatment adherence under trial conditions. This study, conducted in routine programmatic settings, showed that M-TRACK (mobile phone reminders plus electronic tracking tool) was feasible and effective in reducing the pre-treatment attrition among people living with HIV. M-TRACK is easily scalable and has the potential to reduce the gap between diagnosis and treatment of HIV in low- and middle-income countries.

## Supplemental material

The supplemental data for this article can be accessed here.
