# Supplementary material for: ‘M-TRACK’ (mobile phone reminders and electronic tracking tool) cuts the risk of pre-treatment loss to follow-up by 80% among people living with HIV under programme settings: a mixed-methods study from Gujarat, India
Source: Glob Health Action. 2018 Feb 27;11(1):1438239. doi: 10.1080/16549716.2018.1438239 (PMC5827770; doi:10.1080/16549716.2018.1438239)
Supplement: Supplementary material [file ZGHA_A_1438239_SM4007.docx]

**Supplement 1 (a). Data Collection Proforma for both districts in pre M-TRACK period (in excel sheet) and for Rajkot district during M-TRACK period**

| **Data variables** | **Sources of data** | **Data collection** |
| --- | --- | --- |
| **Part 1** | From HIV testing register available at HIV testing centres | Data to be entered by HIV testing center staff (counselor) |
| Serial Number |  |  |
| Line list number with district code |  |  |
| District |  |  |
| Sex (1. Male 2. Female 3. TS/TG) |  |  |
| Age (in years) |  |  |
| Marital status (1. Married 2. Single 3.Divorse/Separate 4.Widowed) |  |  |
| Education (1. Illiterate 2.primary 3.secondary 4.graduate & above) |  |  |
| Occupation (1. Daily Wages 2. Salaried 3. Business 4. House Wife 5. Retired 6. Student 7. Other) |  |  |
| Type of risk behaviour  (1.Heterosexual  2.Homosexual  3.H/O Blood transfusion  4.H/O infected syringe  5.Parent to child  6.Not specified  7.IDU  9.Not recorded) |  |  |
| Phone number |  |  |
| PID number |  |  |
| Date of detection |  |  |
| Name of ART centre referred |  |  |
| **Part 2** | From Pre ART register available at ART centre | Data to be filled by ART staff |
| Registered at ART center (1. Yes 2. No) |  |  |
| If yes then only following details to be filled: |  |  |
| Date of Registration |  |  |
| Pre-ART registration number |  |  |
| ART initiated (1.Yes 2. No) |  |  |
| CD4 count |  |  |
| WHO clinical stage |  |  |

**Supplement 1 (b). Data Collection Proforma for Vadodara district during M-TRACK period**

| **Data variables** | **Sources of data** | **Data collection** |
| --- | --- | --- |
| **Part 1** | From HIV testing register available at HIV testing centres | Data to be entered in google spreadsheet by HIV testing center staff (counselor) |
| Serial Number |  |  |
| Line list number with district code |  |  |
| District |  |  |
| Sex (1. Male 2. Female 3. TS/TG) |  |  |
| Age (in years) |  |  |
| Marital status (1. Married 2. Single 3.Divorse/Separate 4.Widowed) |  |  |
| Education (1. Illiterate 2.primary 3.secondary 4.graduate & above) |  |  |
| Occupation (1. Daily Wages 2. Salaried 3. Business 4. House Wife 5. Retired 6. Student 7. Other) |  |  |
| Type of risk behaviour  (1.Heterosexual  2.Homosexual  3.H/O Blood transfusion  4.H/O infected syringe  5.Parent to child  6.Not specified  7.IDU  9.Not recorded) |  |  |
| Availability of phone (mobile)  1. Yes 2. No |  |  |
| Phone number |  |  |
| Functional mobile number  1. Yes 2. No |  |  |
| PID number |  |  |
| Date of detection |  |  |
| Name of ART centre referred |  |  |
| **Part 2** | From Pre ART register available at ART centre | Data to be filled by ART staff in google spreadsheet |
| Registered at ART center (1. Yes 2. No) |  |  |
| If yes then only following details to be filled: |  |  |
| Date of Registration |  |  |
| Pre-ART registration number |  |  |
| ART initiated (1.Yes 2. No) |  |  |
| CD4 count |  |  |
| WHO clinical stage |  |  |
| **Part 3**  **(to be filled if patient is not registered after one week of diagnosis)** | PLHIV interview over phone call by HIV testing centre staff | Data to be entered by HIV testing center staff (counselor) in google spreadsheet |
| 1^st^ mobile phone reminder at end of 1^st^ week  (1. Unable to call  2. Able to call but unable to contact  3. Able to contact and patient informed that he has been registered  4. Able to contact and patient informed that he has not been registered  7. Not Applicable) |  |  |
| 2^nd^ mobile phone reminder at end of 2^nd^ week  (1. Unable to call  2. Able to call but unable to contact  3. Able to contact and patient informed that he has been registered  4. Able to contact and patient informed that he has not been registered  7. Not Applicable) |  |  |
| 3^rd^ mobile phone reminder at end of 3^rd^ week  (1. Unable to call  2. Able to call but unable to contact  3. Able to contact and patient informed that he has been registered  4. Able to contact and patient informed that he has not been registered  7. Not Applicable) |  |  |
| 4^th^ mobile phone reminder at end of 4^th^ week  (1. Unable to call  2. Able to call but unable to contact  3. Able to contact and patient informed that he has been registered  4. Able to contact and patient informed that he has not been registered  7. Not Applicable) |  |  |

**Supplement 2. Guide for Group interview with health care providers (Counsellors)**

After a brief introduction to the participants regarding the purpose of the discussion, the moderator took will take informed consent for the discussion and record keeping. Consent will also be requested for audio recording.

1. M-TRACK has been initiated in Vadodara district. What do you all think about M-TRACK? [Probe: positive and negative points, give examples]
2. Why do you all think M-TRACK is effective/not effective in reducing loss to follow-up? [Probe: local level, patient-level]
3. What are the problems faced by you all while implementation of M-TRACK?

[Probe: local level, patient-level]

1. Please tell us about some of the factors which facilitated the implementation of M-TRACK in your centre?
2. Were there any reasons regarding unable to contact PLHIV by mobile phone?

[Probe: local level, patient-level]

1. What are the operational issues involved in this implementation: both at your (provider) level and patient level?
2. How can we further improve the implementation of M-TRACK? Please give some suggestions for its improvement.
3. Additional remarks, if any?

Discussion ended by acknowledging the time spared by all the participants from his/her busy schedule. A summary of the notes taken was read aloud to confirm the same from all the participants.

**Supplement 3. Key informant (Program Managers) Interview guide**

**Name of the participant:**

**Designation: Date of Interview:**

**Interview start / end time: Name of the Interviewer:**

After a brief introduction to the participant regarding the purpose of the interview, the principal investigator (PI) will take informed consent for the interview.

1. M-TRACK has been initiated in Vadodara district. What do you think about M-TRACK? [Probe: positive and negative points, give examples]
2. Why do you think M-TRACK is effective/not effective in reducing loss to follow-up? [Probe: programmatic, patient-level]
3. What are the problems faced by you while implementation of M-TRACK?

[Probe: programmatic, local level, patient-level]

1. Is there any delay in M-TRACK implementation? If yes, why is it so?

[Probe: programmatic, patient-level]

1. Were there any reasons regarding unable to contact PLHIV by mobile phone?

[Probe: programmatic, patient-level]

1. What are the operational issues involved in this implementation: both at provider level and patient level?
2. How can we further improve the implementation of M-TRACK in other districts of the state?
3. Additional remarks, if any?

PI will complete the interview by acknowledging the time spared by the participant from his/her busy schedule. He will also give a summary of the notes taken and confirm the same from the participant.

**Supplement 4. Interview guide for telephonic interviews with People living with HIV (PLHIV) who have received mobile phone reminders**

**Name of the participant:**

**Age: Gender:**

**Date of Interview: Interview start / end time:**

**Name of the Interviewer:**

After a brief introduction to the participant regarding the purpose of the interview, the principal investigator (PI) will take verbal informed consent for the interview.

1. Did you get calls from HIV testing centre staff for giving reminder to get registered at ART centre?
2. What were the difficulties / problems faced by you during such calls from them? [Probe: any negative points, examples]
3. What were the good things about them? [Probe: for any help offered, other options given, counselling]
4. Would you recommend such mobile phone reminders to other patients like you?
5. Can you tell us about any other method more appropriate than mobile phone reminders to you?
6. Additional remarks, if any?

PI will complete the interview by acknowledging the time spared by the participant from his/her busy schedule. He will also give a summary of the notes taken and confirm the same from the participant.

**Supplement 5.** **Client related and staff related challenges and solutions as perceived by health care providers for M-TRACK implementation in Vadodara, Gujarat during October-December 2016**

| **Themes** | | **Codes** | **Verbatim quotes** | | **Suggested solutions** | |
| --- | --- | --- | --- | --- | --- | --- |
| Client related challenges | | Preference for call timings out of office hours | “Many times patient may not be able to talk during office hours and they suggest us to call in the evening. Now, we cannot come to the office to call our clients in the evening” | | Provision of mobile phones to counsellors | |
|  | | Verbal abuse/threats | “Some clients verbally abuse us when we call them and disconnect the call. Some even threaten to lodge police complaint and dragging us in court case. So, how to ensure our security when there are such instances?”  “If there are any legal actions initiated against us, we may be put at risk of losing our job.” | | Consent for phone calls to be taken along with HIV testing consent during pre-test counselling  There should be a mention in the appointment orders that counsellors are authorized to call PLHIV over phone  Provision of protection to counsellor in case of legal action | |
|  | Language barrier | | | “It is especially difficult when patient is coming from Madhya Pradesh. We have to explain in detail. Sometimes it may take more than 4 phone calls.” | | Capacity building of the counsellors for counselling in Hindi language |
|  | Patient Confidentiality | | | “When the patient has to be contacted through someone else in the family (when patient does not own the phone), we have to first explain in detail to reach the client. In such cases, we may have to break the confidentiality.” | | Consent for phone calls (through the identified contacts) to be taken along with HIV testing consent during pre-test counselling |
|  | No phone with client | | | “Some of our clients are very poor and they do not possess mobile phones.”  “I am from a tribal area. Some of our clients do not just have money let alone mobile phones. So there is absolutely no possibility of communication.” | | To contact using the phone numbers of family members, caretakers or frontline health workers residing close to PLHIV’s residence  To share the contact details of the counsellor with the client and ask them to call up, if required |
| Staff related challenges | Disturbance in personal life | | | “Some clients prefer to talk on Sunday. In that case we have to call them on Sunday. Sometimes we also have to go for home visit on Sunday. If required we keep the office open on Sundays, otherwise we meet the client at any other convenient public place.”  “When patient gets our mobile number, they call us any time even at night which creates problems in our family” | | PLHIV need to be counselled at the time of diagnosis that they should avoid calling outside office hours and on holidays, except in emergencies |
|  | No regular intimation by ART staff | | | “We send the filled Annexure 2 (excel sheet) every week to ART centre. But we do not receive it back on time.”  “We have to enquire whether our patient has reached or not by calling the ART centre every week.” | | Regular monitoring on daily basis by district supervisor and on weekly basis by state officer |
|  |  | | |  | |  |

HIV = human immunodeficiency virus, PLHIV = People living with the human immunodeficiency virus,

M-TRACK= mobile phone reminders and electronic tracking tool

**Supplement 6.** **Phone related, M-TRACK tool related and programmatic challenges and solutions as perceived by health care providers in Vadodara, Gujarat during October-December 2016**

| **Themes** | **Codes** | **Verbatim quotes** | **Suggested solutions** |
| --- | --- | --- | --- |
| Phone related | Non functional landline | “Almost all centres have landline phones, but they are non functional at most of the places.” | Provision of mobile phone and call allowance |
|  | Inconvenient to use the landline phone | “There is always ambient noise. The office staff might be talking aloud or even laughing over their internal discussion. Because of this patient might feel stigmatized. Overall this makes the counselling difficult.”  “Sometimes it takes 10-15 minutes discussion with the client. If we continue this long conversation, the admin staff generally does not like it.”  “We have to make a note in the phone register when we use office phone. We also have to write the purpose of calling. It is not a comfortable environment.” | Provision of mobile phone and allowance |
|  | Confidentiality issue with landline | “We have to address PLHIV by their name, so the other staff members of the hospital also get to know serostatus of that client.”  “We are uncomfortable in their [other office staff] presence while discussing some sensitive issues with the client” | Provision of talk time (on mobile) allowance to counselor |
| M-TRACK Tool related | No real time entry due to internet connectivity issues | “Not all centres have internet connection. At places where the connection is available, usually there are problems of connectivity. So we are not able to data in real time in the google spreadsheet. So, we complete the excel sheet [offline] and send it to ART centre [by email]” | Internet allowance to providers  Development of a comprehensive software for “PLHIV- continuum of care” from ICTC to ART |
|  | No dedicated computer available | “It was not possible to fill up google spreadsheet because the staff members using computer do not allow us to use it. They already have large load of (online) data entry for other health programmes” | Data entry using mobile phone  Special 1-day training for counsellors to fill spreadsheet in mobile phone |
|  |  |  |  |
| Programme related | Lack of monitoring | “Mobile phone reminders is a good approach for reducing LFU from ICTC to ART center. But, at programmatic level, I feel it is difficult to sustain without regular follow up mechanism from state level (GSACS).” | Regular monitoring on daily basis by district supervisor and on weekly basis by state officer |
|  | No provision of mobile phone, call allowance and internet allowance | “We had to bear the call charges.”  “We should get the reimbursement of the call charges. Otherwise the motivation to work will be affected.”  “RNTCP provides mobile allowance why can’t we think of the same in this programme?” | Provision of mobile phone, call allowance and internet allowance by GSACS |

HIV = human immunodeficiency virus, PLHIV = People living with the human immunodeficiency virus,

M-TRACK= mobile phone reminders and electronic tracking tool

ICTC=Integrated Counseling and Testing Centre, ART=Anti Retroviral Therapy, GSACS=Gujarat State AIDS Control Society, RNTCP=Revised National Tuberculosis Control Programme, LFU=Loss to follow up

**Supplement 7: Content of telephonic call by HIV testing center staff to patient**

|  |  |  |
| --- | --- | --- |
|  | Greetings, Good morning/afternoon! Am I speaking to _______ (name of PLHIV)? |  |
|  |  | |
|  |  |  |
| If Yes |  | If No |
|  |  |  |
| I am a staff from HIV testing centre (name of staff and center) |  | If same gender, then "I am his/her friend, and I need to talk to him" If opposite gender, then "I am staff from hospital, I want to talk to him/her" |
|  |  |  |
| Are you free? Can I talk to you for some time? |  |  |
|  |  |  |
| Did you go to the ART centre (name of centre)? |  | If No |
|  |  |  |
| If Yes |  |  |
|  |  | Why you have not reached? |
|  |  | Any other Problems/Challenges? |
| Where? |  | Do you need any help? |
| When? ART started or not? |  | Try to address the problems/reasons by proper counselling |
|  |  | Give option about other ART centre |
|  |  | Rule out Financial problems |
|  |  | Create awareness about risk of disease |
|  |  |  |
